# Pitfalls in Valganciclovir Prophylaxis Dose Adjustment Based on Renal Function in Kidney Transplant Recipients

**DOI:** 10.3389/ti.2024.12712

**Published:** 2024-05-09

**Authors:** Nathalie Hammer, Linard Hoessly, Fadi Haidar, Cédric Hirzel, Sophie de Seigneux, Christian van Delden, Bruno Vogt, Daniel Sidler, Dionysios Neofytos

**Affiliations:** ^1^ Service of Nephrology and Hypertension, Inselspital, Bern, Switzerland; ^2^ Swiss Transplant Cohort Study (STCS), University Hospital Basel, Basel, Switzerland; ^3^ Service of Nephrology, Geneva University Hospitals (HUG), Geneva, Switzerland; ^4^ Service of Infectious Diseases, Inselspital, Bern, Switzerland; ^5^ Transplant Infectious Diseases Unit, Service of Infectious Diseases, HUG, Geneva, Switzerland

**Keywords:** cytomegalovirus, valganciclovir, renal function, kidney transplantation, dosing

## Abstract

Valganciclovir (VGC) is administered as prophylaxis to kidney transplant recipients (KTR) CMV donor (D)+/recipient (R)− and CMV R+ after thymoglobulin-induction (R+/TG). Although VGC dose adjustments based on renal function are recommended, there is paucity of real-life data on VGC dosing and associations with clinical outcomes. This is a retrospective Swiss Transplant Cohort Study-embedded observational study, including all adult D+/R− and R+/TG KTR between 2010 and 2020, who received prophylaxis with VGC. The primary objective was to describe the proportion of inappropriately (under- or over-) dosed VGC week-entries. Secondary objectives included breakthrough clinically significant CMV infection (csCMVi) and potential associations between breakthrough-csCMVi and cytopenias with VGC dosing. Among 178 KTR, 131 (73.6%) patients had ≥2 week-entries for the longitudinal data of interest and were included in the outcome analysis, with 1,032 VGC dose week-entries. Overall, 460/1,032 (44.6%) were appropriately dosed, while 234/1,032 (22.7%) and 338/1,032 (32.8%) were under- and over-dosed, respectively. Nineteen (14.5%) patients had a breakthrough-csCMVi, without any associations identified with VCG dosing (*p* = 0.44). Unlike other cytopenias, a significant association between VGC overdosing and lymphopenia (OR 5.27, 95% CI 1.71–16.22, *p* = 0.004) was shown. VGC prophylaxis in KTR is frequently inappropriately dosed, albeit without meaningful clinical associations, neither in terms of efficacy nor safety.

## Graphical Abstract

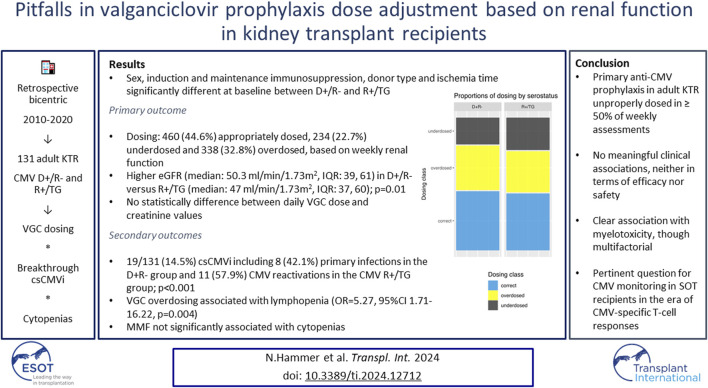

## Introduction

Clinically significant cytomegalovirus infections (csCMVi) are one of the most common complications after a solid organ transplant (SOT), depending primarily on the donor/recipient (D/R) CMV serology status and the net state of immunosuppression [1]. Prophylactic strategies have included the administration of (val)ganciclovir in high-risk patient populations, for 3 months in CMV R+ receiving induction immunosuppression with thymoglobulin (R+/TG) or 6 months in CMV D+R- [2–6]. Orally administered VGC is administered at a dose of 900 mg daily for prophylaxis in patients with normal renal function [3]. Due to low protein binding, VGC is renally eliminated via both glomerular filtration and active tubular secretion and requires dose adjustment based on renal function [7]. Adjusted VGC dosing has been proposed, although there are no good data to adequately correlate VGC dose with plasma concentrations and therapeutic drug monitoring (TDM) is rarely available and not well validated [8]. Lack of evidence is even more problematic in patients requiring continuous renal replacement therapy or hemodialysis for delayed graft function (DGF) [9]. Despite lack of adequate evidence, VGC dose adjustments based on renal dysfunction are made in most transplant centers worldwide, predominately to prevent neutropenia [10]. However, lower dose administration may lead to decreased drug concentrations, resulting in breakthrough csCMVi and/or (val)ganciclovir resistance selection [8]. Furthermore, in kidney transplant recipients (KTR) renal function may change over time, particularly early post-transplantation, necessitating frequent monitoring and adjustment of VGC dosing [11]. The latter may be particularly cumbersome and prone to mistakes, for those KTR discharged with still impaired renal function and renally dosed VGC requiring close and frequent ambulatory follow-up.

We hypothesized that VGC dosing is not properly adjusted to renal function based on established recommendations, due to lack of patient monitoring particularly on an outpatient basis, potentially leading to higher rates of breakthrough csCMVi or VGC associated toxicities during the first 3–6 months post-transplant. We aim to describe the proportion of VGC primary CMV prophylaxis weekly doses, that are either under- or over-dosed according to renal function.

## Materials and Methods

This was a two-center retrospective observational study conducted at the University Hospitals of Geneva and Bern, in Switzerland. All adult (>18-year-old) CMV D+R- or CMV R+/TG KTR, who received a kidney transplant between 1st January 2010 and 31st December 2019, had a follow-up of 1-year post-transplant, and who had signed an informed consent form to participate in the Swiss Transplant Cohort Study (STCS) were included. The study was approved by the responsible Ethics Committees (2022-00959) and the STCS (FUP 197/2022).

### Objectives

The primary objective was to describe the proportion of inappropriately dosed VGC primary CMV prophylaxis weekly entries. The following secondary objectives were studied: 1) the incidence of breakthrough csCMVi, 2) potential associations between breakthrough csCMVi and VGC dosing, and 3) the incidence of cytopenias and potential associations with VGC dosing considering the potential myelosuppressive effect of VGC. All objectives were assessed during the first 3 and 6 months in CMV R+/TG and CMV D+R− KTR, respectively.

### Definitions

Valganciclovir dosing was based on published guidelines [3, 12]. Briefly, VGC prophylaxis was considered appropriate if dosed at 900 mg daily in patients with an estimated glomerular filtration rate (eGFR) ≥60 mL/min/m^2^, and reduced to 450 mg daily, every 48 h, and twice weekly in patients with eGFR at 40–59 mL/min/m^2^, 25–39 mL/min/m^2^, and 10–24 mL/min/m^2^, respectively (Supplementary Dosing Schema) [11]. There is no recommendation for an eGFR <10 mL/min/m^2^. Inappropriate VGC dosing included underdosing and overdosing, defined as any dose below and above the predetermined eGFR ranges, respectively. Inappropriate dosing can be influenced by an early graft dysfunction, such as a delayed graft function (DGF), defined as an acute kidney injury (AKI) which occurs in the first week after transplantation or a primary non function (PNF), defined as permanent lack of graft function from the time of transplantation, both requiring a dialysis treatment [13, 14]. CMV infection and disease were defined based on international guidelines [15]. csCMV infection (csCMVi) was defined as any CMV infection (asymptomatic CMV DNAemia, CMV viral syndrome, probable or proven CMV disease) for which anti-CMV preemptive or targeted treatment was initiated. Breakthrough csCMVi was defined as any CMV infection/disease diagnosed while patients were receiving prophylaxis with VGC [16]. Cytopenias were defined based on laboratory thresholds used in both centers, which defined leucopenia as a leucocyte count <3 G/L, neutropenia as an absolute neutrophil count (ANC) < 1.5 G/L, lymphopenia as an absolute lymphocyte count (ALC) <1 G/L, and thrombocytopenia as platelet count <150 G/L.

### Institutional Practices

Primary CMV prophylaxis with VGC was administered for 6 and 3 months post-transplant in CMV D+R− and CMV R+/TG KTR, respectively, in both centers. Plasma measured CMV DNAemia was monitored by quantitative polymerase chain reaction (qPCR). To facilitate prescription and avoid potential mistakes, it has been established based on institutional protocol to perform weekly CMV DNAemia in all CMV D+R− and CMV R+ patients, despite or not primary anti-CMV prophylaxis is administered. In Geneva, CMV PCR was performed on plasma with the COBAS^®^ 6800 test (Roche Diagnostics, Indianopolis, United States), with a level of detection (LOD) and quantification (LOQ) of 21 IU/mL and 25 IU/mL, respectively. In Bern, CMV PCR was performed on plasma by an in-house test (Roche Diagnostics, LightCycler z480 II, Indianopolis, United States) using copies/mL with a LOQ starting from 500 copies/mL. Results in copies/mL at one center were converted to IU/mL, using the 1 IU/mL = 0.91 copies/mL equivalence formula [17, 18]. The primers and probes were synthesized by Eurofins. The accepted threshold to initiate therapy was >1,000 IU/mL in both centres.

### Data Collection

The following data were retrieved from the STCS database, including demographics (age, sex, and body mass index), baseline comorbidities (diabetes mellitus, hypertension, coronary heart disease and smoking), hemodialysis requirement and transplantation-related variables, such as induction and maintenance immunosuppressive regimens, donor type, and cold ischemia time. Renal function, assessed as creatinine and eGFR, calculated using the Chronic Kidney Disease Epidemiology Collaboration equation from 2012 (CKD-EPI 2012), and other laboratory values such as leucocytes, ANC, ALC, and platelet count were collected through patient electronic charts. VGC dosing, renal function and blood cell count variables, and CMV DNAemia were collected weekly until the end of VCG prophylaxis administration. For patients with breakthrough csCMVi data collection was stopped on the day of the infection diagnosis. Hence, results are presented per patient for the baseline patient characteristics and per weekly entries for VGC dosing and csCMVi. Project data collected based on the Case Report Form (CRF) were transferred to electronical records in Redcap^®^ prior to analysis. For the Bern population, source documents of laboratory analyses were stored on SharePoint and individual values were automatically imported to Redcap^®^. All weekly data entries were restricted to entries where the VGC dosing was defined, and further restricted to follow-up week 12 and 24 for CMV R+/TG and CMV D+R− KTR, respectively.

### Statistical Analysis

Quantitative variables are presented as medians (with interquartile ranges, IQR). Qualitative variables are presented as numbers and percentages. To compare patients across the serostatus group, we used the Student’s *t*-test (or Mann-Whitney-U test) and for more than two groups, we used ANOVA (or Kruskal–Wallis test). For categorical variables Fisher’s exact test was used. Statistical significance was assumed for *p* < 0.05 and all tests were two-tailed. Longitudinal data were reported in week-entries, according to the follow-up of patients. In order to ensure a minimal number of weekly entries of VGC prophylaxis dosing classification, patients had to have at least 2 weekly entries with VGC dosing and eGFR in the first 3 and 6 months for R+/TG and D+R−, respectively. Patients with insufficient week entries were excluded from outcome analysis. To investigate the effect of CMV serostatus on csCMV, we performed a cause-specific Cox proportional hazards model. Competing events were a new transplantation, death, or loss to follow-up. Fisher’s exact test was used to compare cumulative incidence of breakthrough csCMVi for associations between VGC dosing and breakthrough csCMVi and myelotoxicity. To explore the effect of overdosing on cytopenia, we used mixed effects logistic regression models, i.e., *generalized linear mixed models*. The *random intercept* included in the model varied among patients, accounting for the variation in measurements for subjects due to multiple measurements over time. We additionally accounted for follow-up week and weekly MMF medication in the models. The analyses were limited to the weeks with complete entries for cytopenia. As the time trend might not be identical for each patient, we did a sensitivity analysis with the same models, but additionally with a random slope for time. All statistical analysis were performed on R Version 4.3.2. Generalized mixed models were fitted using the R package “Ime4”. The package “ggplot2” was used for visualization.

## Results

### Characteristics of the Study Population

**FIGURE 1:**
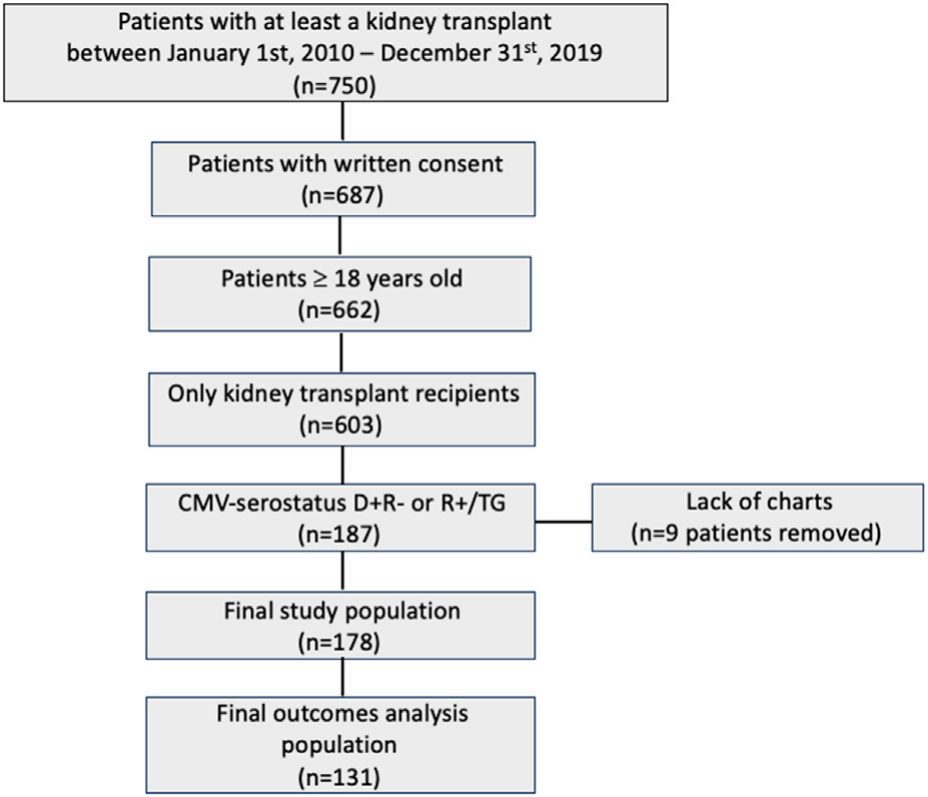
Study population Flowchart.

From 750 KTR, 178 patients fulfilled the inclusion criteria and were included in the study (Figure 1). There were 114 patients (64%) recruited in Geneva and 64 (36%) in Bern. The median age was 55.3 years (IQR 42.7, 63.7), most patients were male (*n* = 122, 68.5%), with a median BMI of 25.5 kg/m^2^ (IQR 23.4, 29.3). Baseline patient characteristics were comparable between CMV R+/TG and CMV D+R− patients, except for sex (male *n* = 46, 59% versus *n* = 76, 76%; *p* = 0.02), cold ischemia time (346 min versus 552 min; *p* = 0.03), immunosuppressive induction treatment by thymoglobulin (*n* = 78, 100% versus *n* = 16, 16%; *p* < 0.001), immunosuppressive maintenance by ciclosporine (*n* = 48, 27%; *p* < 0.001), tacrolimus (*n* = 46, 46%; *p* < 0.001) and MMF (*n* = 102, 57.3%; *p* < 0.001), and the type of donor (*n* = 87, 48.9% DBD versus *n* = 67, 37.6% living versus *n* = 24, 13.5% DCD); *p* < 0.001; Table 1).

**TABLE 1:** Baseline patient characteristics.

	D+/R- *n* = 100 (%)	R+/TG *n* = 78 (%)	Total *n* = 178 (%)	*p*-value
**Demographics**				
Sex, Male	76 (76)	46 (59)	122 (68.5)	0.02
Age (years) median (IQR)	56.4 (42.2, 66.5)	54.6 (43.1, 61.7)	55.3 (42.7, 63.7)	0.51
BMI (kg/m^2^) median (IQR)	25.5 (23.2, 28.1)	25.9 (23.5, 29.5)	25.5 (23.4, 29.3)	0.55
Weight (kg) median (IQR)	77 (65.4, 89.8)	74.1 (61, 87.4)	76 (63.3, 88.8)	0.19
**Comorbidities**				
Hypertension	82 (82)	71 (91)	153 (86)	0.13
Diabetes	14 (14)	14 (17.9)	28 (15.7)	0.54
Coronary heart disease	16 (16)	20 (25.6)	36 (20.2)	0.13
Smoking^a^	15 (15.5)	7 (9.2)	22 (12.7)	0.26
**Etiologies of kidney disease**				0.18
Glomerulonephritis	25 (25)	14 (17.9)	39 (21.9)	
Glomerulosclerosis	15 (15)	23 (29.5)	38 (21.3)	
ADPKD	21 (21)	13 (16.7)	34 (19.1)	
Diabetes	9 (9)	10 (12.8)	19 (10.7)	
Previous graft failure	6 (6)	6 (7.7)	12 (6.7)	
Reflux/pyelonephritis	3 (3)	4 (5.1)	7 (3.9)	
Congenital	4 (4)	1 (1.3)	5 (2.8)	
Interstitial nephritis	4 (4)	0	4 (2.2)	
Other^b^	13 (13)	9 (11.5)	22 (12.4)	
**Induction immunosuppression**				
Basiliximab^c^	82 (82)	12 (15.4)	94 (52.8)	<0.001
Thymoglobulin	16 (16)	78 (100)	94 (52.8)	<0.001
**Maintenance immunosuppression**				<0.001
Ciclosporine	37 (37)	11 (14.1)	48 (27)	<0.001
Tacrolimus	46 (46)	58 (74.4)	104 (58.4)	<0.001
MMF	69 (69)	33 (42.3)	102 (57.3)	<0.001
mTOR	3 (3)	1 (1.3)	4 (2.2)	0.63
Cold ischemia time (min) median (IQR)	346 (100.8, 580.3)	552 (96.8, 787.5)	391 (100.3, 665.5)	0.03
Previous renal graft	12 (12)	11 (14.1)	23 (12.9)	0.68
**Number of previous grafts**				0.93
1	88 (88)	67 (85.9)	155 (87.1)	
2	10 (10)	9 (11.5)	19 (10.7)	
>2	2 (2)	2 (2.6)	4 (2.2)	
Dialysis prior to transplant	64 (64)	47 (60.3)	111 (62.4)	0.61
**Dialysis type prior to transplant**				0.48
HD	49 (76.6)	39 (83)	88 (79.3)	
PD	15 (15.5)	8 (17)	22 (12.7)	
**Donor type**				<0.001
DBD	52 (52)	35 (44.9)	87 (48.9)	
Living	43 (43)	24 (30.8)	67 (37.6)	
DCD	5 (5)	19 (24.4)	24 (13.5)	
**Donor**				
Sex, Female	58 (58)	43 (55.1)	101 (56.7)	0.76
Age (Years) Median (IQR)	57 (47.8, 64.3)	53.5 (44.3, 61)	55 (46, 63)	0.12
**Kidney dysfunction post-transplant**				0.29
DGF	21 (21)	24 (30.8)	45 (25.3)	
PNF	3 (3)	1 (1.3)	4 (2.2)	

ADPKD, autosomal dominant polycystic kidney disease; BMI, body mass index; DBD, donor after brain death; DCD, donor after cardiac death; DGF, delayed graft function; HD, hemodialysis; MMF, mycophenolate mofetil; mTOR, mammalian target of rapamycin; PD, peritoneal dialysis; PNF, primary non function.

^a^ There were five missing values.

^b^ Other etiologies included nephrocalcinosis, thrombotic microangiopathy, acute kidney injury post sepsis, eclampsia, cortical necrosis or unknown.

^c^ Some patients induced with basiliximab could receive a supplemental treatment with thymoglobulins due to DGF or acute rejection.

### Valganciclovir Dosing

**FIGURE 2:**
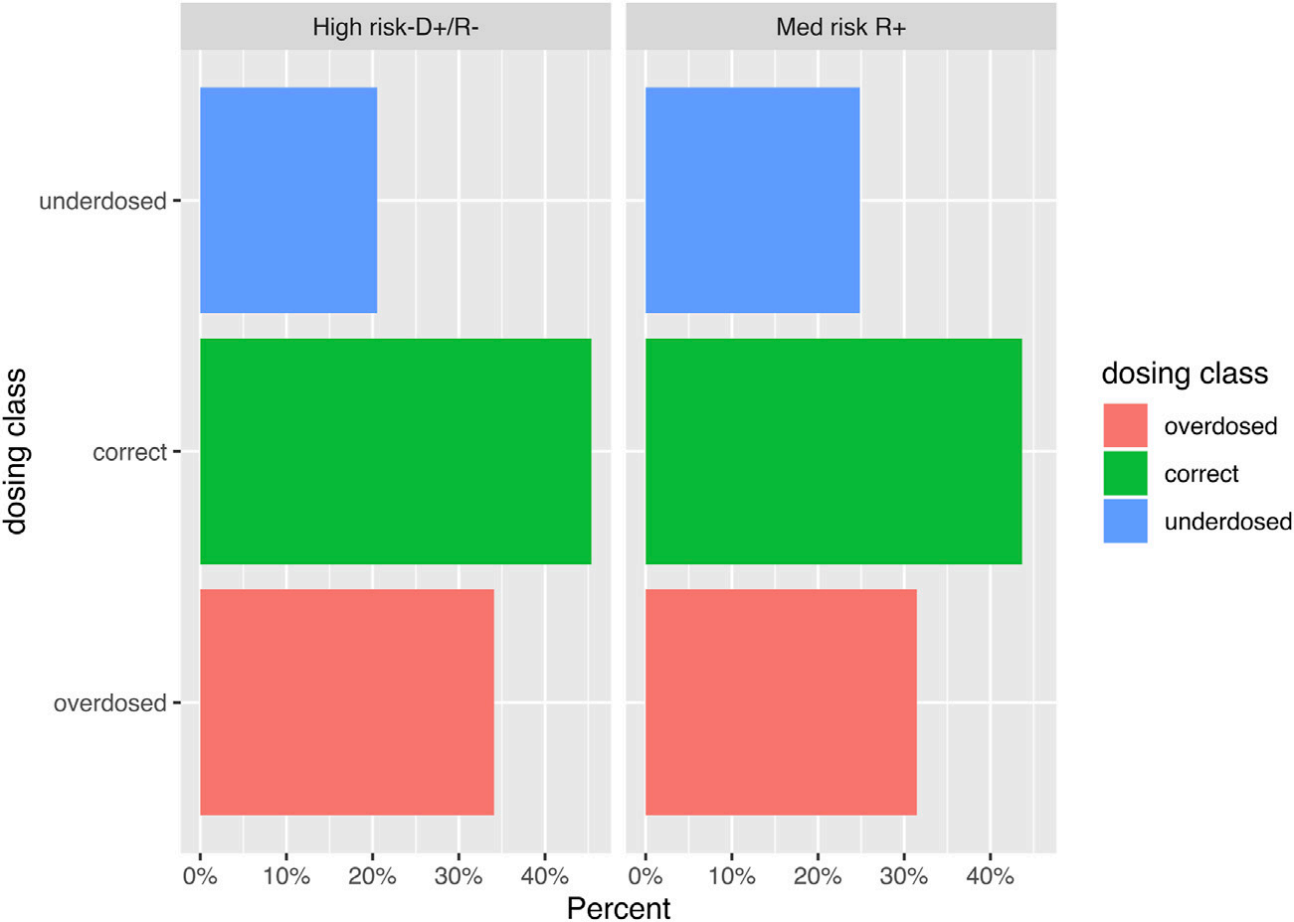
Bar graph visualizing the distribution of the weekly valganciclovir dosing entries (*n *= 1,032) according to the CMV serostatus of donors and recipients. The difference in proportion of entries was highest for underdosed entries, where 20.7% of the weekly entries of CMV donor (D)+ recipient (R)- kidney transplant recipients were underdosed versus 24.8% of the entries of CMV R+ patients.

Among 178 patients, 131 patients (73.6%) had at least 2 week entries for the longitudinal data of interest and were included in the outcome analysis (Figure 1). Their baseline characteristics are reported in Supplementary Table S1. There were 1,032 weekly VGC dose entries for 131 patients, for a median of 6 (IQR 3, 9) entries per patient over the entire prophylaxis period. Overall, 460 (44.6%) were appropriately dosed, while 234 (22.7%) and 338 (32.8%) were under- and over-dosed, respectively, based on the recorded weekly renal function values (Figure 2). Daily VGC dose (*p* = 0.09) and creatinine value (*p* = 0.56) were not significantly different among D+/R− and R+/TG. However, eGFR was higher (median: 50.3 mL/min/1.73 m^2^, IQR: 39, 61) in D+/R− versus R+/TG (median: 47 mL/min/1.73 m^2^, IQR: 37, 60; *p* = 0.01) patients (Table 2). Overall, inappropriate dosing was similar during the first 4 weeks (225/390, 57.7%) with later (>4 weeks, 347/642, 54%, *p* = 0.27) post-transplant. In contrast, inappropriate dosing was more frequent among R+/TG (133/214, 62.1%) than D+/R− (92/176, 52.3%, *p* = 0.05) during the first 4 weeks post-transplant compared to later. Weekly VGC prophylaxis dosing according to renal function is described in detail in Supplementary Table S2. Dose appropriateness did not significantly differ between D+R− (241/532, 45.3%) and R+/TG (219/500, 43.8%; *p* = 0.66). In contrast, VGC was less likely to be appropriately dosed in Geneva (329/767, 42.9%) compared to Bern (131/265, 49.4%; *p* < 0.001).

**TABLE 2:** Valganciclovir dose assessment as appropriate, under- or over-dosing based on the CMV donor/recipient status.

	Valganciclovir dose assessment			
	D+/R- *n* = 532 (%)	R+/TG *n* = 500 (%)	Total *n* = 1,032 (%)	*p*-value
Appropriately dosed	241 (45.3)	219 (43.8)	460 (44.6)	0.28
Overdosed	181 (34)	157 (31.4)	338 (32.8)	
Underdosed	110 (20.7)	124 (24.8)	234 (22.7)	
Daily VGC dose in mg, median (IQR)	450 (450, 900)	450 (450, 450)	450 (450, 900)	0.09
Creatinine (umol/L), median (IQR)	133 (108, 160)	130.5 (106, 160)	131 (107, 160)	0.56
eGFR (ml/min/1.73 m^2^) median (IQR)	50.3 (39, 61)	47 (37, 60)	49 (38, 60)	0.01

D Donor, R Recipient, IQR, interquartile range; VGC, valganciclovir.

### Breakthrough csCMV Infections

Of the 131 patients, 19/131 (14.5%) had breakthrough csCMVi. By comparing among serostatus, there were 8 (42.1%) primary infections in the D+R− group and 11 (57.9%) CMV reactivations in the CMV R+/TG group (*p* < 0.001), but no statistically difference in term of symptomatology presentation, *p* = 0.06; (Supplementary Table S3). Comparisons of the weekly VGC dose performed between patients with and without a breakthrough csCMVi, taking into consideration the appropriateness of all weekly VGC doses for the former and those during the 2 weeks prior to the breakthrough csCMVi in the latter group, respectively, did not show any difference between the two groups. Similarly, there was no statistically significant difference in the rate of breakthrough csCMVi between patients with underdosed weekly VGC doses (3/20, 15%) compared to those patients without VGC underdosing (226/952, 23.7%; *p* = 0.44). In multivariable Cox analysis, CMV R+/TG KTR had essentially the same risk to develop a csCMVi compared to D+R− [HR 1.02, 95% CI (0.32–3.30), *p* = 0.97], even when adjusting for maintenance immunosuppression^1^.

### Cytopenia

**FIGURE 3:**
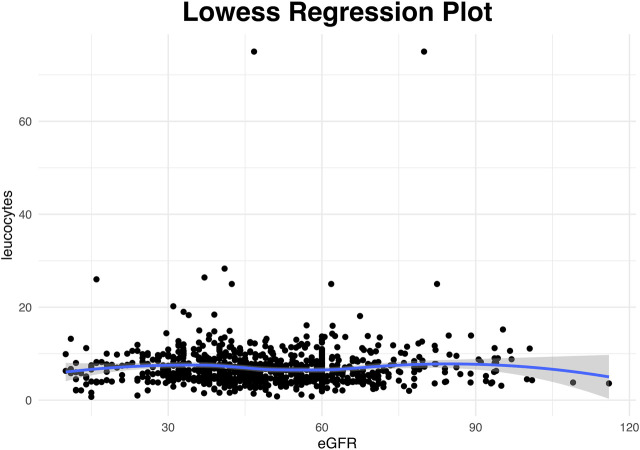
Scatterplot for the available weekly entries demonstrating the association between renal function presented as glomlular filtration rate (eGFR) and leucocyte counts trough a lowess smoother. (Analysis restricted to entries from the second follow-up week onwards with four extreme values that had eGFR above 180 or leucocytes above 200 manually removed). The rather horizontal nonlinear line indicates that there was no association between eGFR and leucocytes.

Leucopenia, neutropenia and thrombocytopenia was reported in a small proportion of tested samples (48/928, 5.2%, 23/735, 3.1%, and 58/880, 6.6%, respectively). In contrast, lymphopenia was observed in more than 2/3 of specimens tested (566/742, 76.3%). There was no statistically significant difference in the proportion of weekly leucopenia, neutropenia, or thrombocytopenia values based on whether VGC was overdosed or not (*p* = 0.63, *p* = 0.48, and *p* = 0.65), respectively. In contrast, lymphopenia was more frequently observed when VGC was overdosed (*p* = 0.01; Table 3 and Figure 3). Considering the potential myelosuppressive effect of VGC and MMF, mixed effects logistic regression models were developed (Table 4). While a significant association between VGC overdosing and lymphopenia (OR = 5.27, 95% CI 1.71–16.22, *p* = 0.004) was shown, there were no significant associations between VGC overdosing and leucopenia (OR = 2.28, 95% CI 0.49–10.48, *p* = 0.29), neutropenia (OR = 2.45, 95% CI 0.28–21.62, *p* = 0.42), and thrombocytopenia (OR = 0.74, 95% CI 0.21–2.65 *p* = 0.64). Similarly, there were no significant associations of MMF with lymphopenia (OR = 1.78, 95% CI 0.21–15.37, *p* = 0.6), leucopenia (OR = 2.83, 95% CI 0.34–23.35, *p* = 0.33), neutropenia (OR = 0.22, 95% CI 0.003–15.10, *p* = 0.48), or thrombocytopenia (OR = 1.40, 95% CI 0.03–57.51, *p* = 0.86). We hypothesized the longer the VGC of MMF administration, the more potent their effect on bone marrow suppression. Hence, we included time post-transplant in follow-up weeks in the model, showing a significant association of follow-up weeks on lymphopenia (OR = 1.16, 95% CI 1.07–1.26, *p* < 0.001), leukopenia (OR = 1.54, 95% CI 1.31–1.80, *p* < 0.001), and neutropenia (OR = 1.38, 95% CI 1.18–1.61, *p* < 0.001). As an identical time trend could not be assumed for every patient, we added a sensitivity analysis with additional random slope for FUP weeks (Supplementary Table S4). It confirmed the significant association of VGC overdosing with lymphopenia (OR = 6.65, 95% CI 1.55–28.56, *p* = 0.011), such as the significant association of follow-up weeks on neutropenia (OR = 1.23, 95% CI 1.11–1.37, *p* < 0.001), while only retaining point effects of the OR above for lymphopenia (OR = 2.35, 95% CI 0.72–7.67, *p* = 0.16) and leukopenia (OR = 1.19, 95% CI 0.87–1.61, *p* = 0.28).

**TABLE 3:** Cytopenias in association with valganciclovir dosing.

	Not overdosed *n* = 694 (%)	Overdosed *n* = 338 (%)	Total *n* = 1,032 (%)	*p*-value
Leucopenia^a^	35/644 (5.4)	13/284 (4.6)	48/928 (5.2)	0.63
Neutropenia^a^	15/528 (2.8)	8/207 (3.9)	23/735 (3.1)	0.48
Lymphopenia^a^	393/534 (73.6)	173/208 (83.2)	566/742 (76.3)	0.01
Thrombocytopenia^a^	39/617 (6.3)	19/263 (7.2)	58/880 (6.6)	0.65

^a^ Available values for leucocyte, neutrophil, lymphocyte, and platelet counts were 48, 23, 566, and 58, respectively for: 644, 528, 534 and 617 not overdosed valgancoclovir; 284, 207, 208, and 263 overdosed valganciclovir.

**TABLE 4:** Predictors of cytopenias on multivariable analysis.

Lymphopenia	OR	95% CI	*p*-value
VGC overdosing	5.27	1.71–16.22	0.004
MMF^a^	1.79	0.21–15.37	0.60
FUP	1.16	1.01–1.26	<0.001
Leucopenia			
VGC overdosing	2.28	0.49–10.48	0.29
MMF^a^	2.83	0.34–23.34	0.33
FUP	1.54	1.32–1.81	<0.001
Neutropenia			
VGC overdosing	2.45	0.27–21.99	0.42
MMF^a^	0.22	0.003–15.78	0.49
FUP	1.38	1.16–1.64	<0.001
Thrombocytopenia			
VGC overdosing	0.74	0.21–2.65	0.64
MMF^a^	1.40	0.03–57.5	0.86
FUP	1.03	0.95–1.13	0.44

FUP, follow up weeks; MMF, mycophenolate mofetil; OR, odds ratio; VGC, valganciclovir, 95% CI, 95% confidence interval.

^a^ Overall, 19/105 MMF treatment durations had a missing stopdate, and the duration was imputed via median.

## Discussion

In this two-center observational study we report that VGC administered as primary anti-CMV prophylaxis in adult KTR is not properly dosed in more than half of weekly assessments during the first months post-transplant, albeit without significant efficacy and safety associations.

Data on the most effective and safest VGC prophylaxis dosing are lacking. Previous studies reported not properly dosed VGC in association with CMV infection and breakthrough csCMVi [19, 20]. Dose adjustments have been proposed to ascertain efficacy, while limiting the potential toxicities associated with higher VCG concentrations, namely, its effect on the bone marrow and associated cytopenias. A lower VGC dosing (450 mg daily for an eGFR ≥60 mL/min/m^2^) has been shown to significantly reduce the incidence of leucopenia and to be cost-effective [21, 22]. For this study, we followed the proposed dose adjustments as shown in the Compendium^®^, an open-access Swiss medication database operated by HCI Solutions SA and regularly updated, which provides short monographs, clear clinical decision support and interaction profiles, and dose adjustments based on renal function of the drugs [23]. Although not identical, the adjustments proposed by the Swiss Compendium are quite similar with other dose adjustment guidelines and recommendations [3]. Rapid changes of renal function are frequent events over the first weeks post-kidney transplantation, ranging from dialysis or pre-dialysis creatinine values to normal values in a few weeks, or the presence of an early graft dysfunction such as DGF or PNF, directly impacting on the dose of multiple medications, including that of VGC, and prompting frequent dose adaptations. In our cohort, 25.3% patients had a DGF while 2.2% had a PNF, which is consistent with the existing literature and could explain why inappropriate VGC dosing was more frequent during the first 4 weeks after transplantation compared to later. That entails close and frequent monitoring of those patients and their renal function, which needs to be assured and organized, particularly once patients are discharged from the hospital and monitored on an outpatient basis. Notably, following a detailed review of weekly renal function and VGC dose assessments during the early period post-KT, our data suggest that VGC is very frequently either over- or under-dosed considering the associated weekly renal function measurement. Although this finding may merely reflect lagging results between bloodwork performed and review by the treating physician and VGC dose adjustment, the number of weekly dissociations between VGC dose and renal function remains quite considerable. Despite an outpatient assessment of KTR once weekly or every other week, it is likely considering the complexity of care of KTR that VGC dosing is not always addressed and hence occasional pitfalls may occur. In fact our data suggest that inappropriate dosing may be even more frequent that we thought, pointing out the need for more careful and intensive monitoring of the patient medication list and doses. The burden and outpatient organization of dose adjustment of medications, including antiinfective agents, in the early post-transplant period in KTR is an area requiring more and consistent studying in the future.

Comparisons of the weekly VGC dose between patients with and without a breakthrough csCMVi was not different between the two groups. Consequently, our hypothesis that VCG under-dosing could have been associated with higher rates of breakthrough csCMVi was not retained despite a global incidence of 14.5%, higher than an incidence between 2.5% and 6.5% reported in the literature [24]. Comparisons of the weekly VGC dose between patients with and without a breakthrough csCMVi was not different between the two groups. Our findings are similar to data reported by Stevens *et al* on the incidence of breakthrough csCMVi among 90 transplant recipients receiving standard (900 mg daily) versus lower (450 mg daily) doses of VGC prophylaxis. There was no significant difference between the two groups with breakthrough csCMVi occurring in a single patient receiving standard VGC dosing and in six patients in the lower VGC dosing group (2.2% versus 13.3%; *p* = 0.11) [16]. Although not definitive, those findings do not call into question the actual VGC dosing recommendations. However, they suggest that an intensive VCG dose monitoring and prompt dose adjustment based on the associated renal function may not be the only and primary determinant of breakthrough csCMVi in KTR during the early post-transplant period, allowing a certain margin of miscalculation without significant clinical efficacy pitfalls. This is an important observation that requires additional research, considering the time and cost investment in renal function and dose adjustment monitoring applied in most transplant centers worldwide. This observation applies in both CMV R+/TG and D+R− patients, as results did not significantly differ based on the D/R serostatus constellation. There was a trend for more csCMVi in patients enrolled in one center, although this could be attributed to different strategies applied in the two centers, including frequency and type of CMV DNAemia monitoring and threshold for preemptive treatment initiation.

Cytopenia is part of the numerous complications occurring post-transplantation and is known to complicate treatment administration in up to 60% of KTR who will experience at least one episode of leucopenia or neutropenia [25]. A meta-analysis found that VGC 900 mg daily was associated with a 3.3 times greater risk of leucopenia [26]. Considering the potential myelosuppressive effect of VGC, its dose requires further adjustments based on renal function results, especially in patients receiving TG for induction after a deceased-donor or in presence of DGF [27]. In our study we found an association between VGC dosing and lymphopenia, which was higher among overdosed patients. Whether lymphopenia could be related to VCG dosing and/or a number of other potential variables, including induction and maintenance immunosuppression, breakthrough csCMVi, or concomitant administration of other medications with potential myelotoxic effect (e.g., MMF, thymoglobulins, trimethoprim/sulfamethoxazole, for example) remains to be better defined [28]. Notably, there were no strong associations between leucopenia or neutropenia and neither VGC overdosing nor MMF administration in our study. This is likely due to the high number of missing values, not allowing us to make any additional meaningful observations between the variables tested, despite the well known myelosuppressive effect of both agents. In fact, when looked at the effect of weekly follow-ups on cytopenias, the only significant association after performing a sensitivity analysis was found by neutropenia. This reflects a potential cumulative effect of those treatments on bone marrow suppression and further highlight a certain dose- and time-effect imputed to a combined myelotoxicity effect of VGC, MMF, and other agents, including thymoglobulin.

Our study has numerous limitations, including its retrospective two-center design, limited number of patients, and even lower number of patients with adequate weekly data to allow for meaningful comparisons and powerful conclusions. In addition, differences in data coding, VGC dosing, outpatient visit frequency, and CMV DNAemia measurement and threshold for preemptive treatment initiation between the two centers might have accounted for higher numbers of csCMVi in one center compared to the other. Finally, eGFR measurements used for renal function assessment were based on the CKD-EPI formula, as recommended by the National Kidney Foundation (NKF) and the American Society of Nephrology (ASN), while the pre-cited guidelines measured eGFR by Cockcroft-Gault equation or Modification of Diet in Renal Disease (MDRD) equation [11, 12, 29, 30].

## Conclusion

Despite its limitations, this bicentric study addresses a pertinent question in the management of post-transplant CMV prophylaxis in the VGC prophylaxis era. Based on our observations, VGC dosing is frequently inappropriate, albeit without meaningful clinical associations, neither in terms of efficacy nor safety. Our findings need to be validated in larger scale studies, in order to better assess the importance of intensive renal function and VGC dose adjustment monitoring in the post-transplant setting. This question remains pertinent, despite the fact that CMV‐specific T‐cell responses and other agents, such as letermovir, may become more prevalent in the monitoring of CMV in SOT recipients in the near future.

## Data Availability

The raw data supporting the conclusion of this article will be made available by the authors, without undue reservation.

## Footnotes

1 Considering the low number of events, we only assessed association with maintenance immunosuppression and serostatus.

## Group members of the Swiss Transplant Cohort Study (STCS)

P. Amico, J.-D. Aubert, V. Banz, S. Beckmann, G. Beldi, C. Berger, E. Berishvili, A. Berzigotti, I. Binet, P.-Y. Bochud, S. Branca, H. Bucher, E. Catana, A. Cairoli, Y. Chalandon, S. De Geest, O. De Rougemont, S. De Seigneux, M. Dickenmann, J.L. Dreifuss, M. Duchosal, T. Fehr, S. Ferrari-Lacraz, C. Garzoni, D. Golshayan, N. Goossens, F.H.J. Halter, D. Heim, C. Hess, S. Hillinger, H.H. Hirsch, P. Hirt, G. Hofbauer, U. Huynh-Do, F. Immer, M. Koller, M. Laager, B. Laesser, F. Lamoth, R. Lehmann, A. Leichtle, O. Manuel, H.P. Marti, M. Martinelli, V. McLin, K. Mellac, A. Merçay, K. Mettler, A. Müller, N.J. Mueller, U. Müller-Arndt, B. Müllhaupt, M. Nägeli, G. Oldani, M. Pascual, J. Passweg, R. Pazeller, K. Posfay-Barbe, J. Rick, A. Rosselet, S. Rossi, S. Rothlin, F. Ruschitzka, T. Schachtner, U. Schanz, S. Schaub, A. Scherrer, A. Schnyder, M. Schuurmans, S. Schwab, T. Sengstag, F. Simonetta, S. Stampf, J. Steiger, G. Stirnimann, U. Stürzinger, C. Van Delden, J.-P. Venetz, J. Villard, J. Vionnet, M. Wick, M. Wilhelm, P. Yerly.

## Abbreviations

ADPKD, autosomal dominant polycystic kidney disease; BMI, body mass index; CMV, cytomegalovirus; csCMVi, clinically significant CMV infection; DBD, donor after brain death; DCD, donor after cardiac death; DGF, delayed graft function; HSC, hematopoietic stem cells; KPD, kidney paired donation; KTR, kidney transplant recipients; MMF, mycophenolate mofetil; mTOR, mammalian target of rapamycin; PAP, primary antiviral prophylaxis; PCR, polymerase chain reaction; PNF, primary non function; SOT, solid organ transplantation; STCS, Swiss Transplant Cohort Study; TG, thymoglobulin; TDM, therapeutic drug monitoring; VGC, valganciclovir.
