# Supplementary material for: Pitfalls in Valganciclovir Prophylaxis Dose Adjustment Based on Renal Function in Kidney Transplant Recipients
Source: Transpl Int. 2024 May 9;37:12712. doi: 10.3389/ti.2024.12712 (PMC11112565; doi:10.3389/ti.2024.12712)
Supplement: Supplementary file 1 [file DataSheet1.docx]

**Supplement dosing schema.** Valganciclovir dosing according to renal function*

| **ClCr (ml/min)** | **Induction therapy** | **Maintenance therapy** |
| --- | --- | --- |
| **≥ 60** | 900 mg (2 tablets) 2x/day | 900 mg (2 tablets) 1x/day |
| **40-59** | 450 mg 2x/day | 450mg 1x/day |
| **25-39** | 450mg 1x/day | 450mg every 2 days |
| **10-24** | 450mg every 2 days | 450mg twice a week |

ClCr: Creatinine clearance.

***Compendium** as dosing reference in our centers (found in the bibliography, item 11).

**Supplement Table 1**. Baseline characteristics of the final outcome analysis population

|  | D+/R-  n=58 (%) | R+/TG  n=73 (%) | Total  n=131 (%) | *p* value |
| --- | --- | --- | --- | --- |
| Demographics |  |  |  |  |
| Sex, Male | 46 (79) | 43 (59) | 89 (68) | **0.02** |
| Age (years)  median (IQR) | 56.4  (46, 67.7) | 54.5  (43.1, 62.2) | 55.1  (44.5, 63.9) | 0.43 |
| BMI (kg/m^2^)  median (IQR) | 24.7  (23, 27.7) | 25.5  (23.5, 29.4) | 25.1  (23.2, 28.8) | 0.24 |
| Weight (kg)  median (IQR) | 75.5  (65.1, 81.8) | 73  (60.5, 86.6) | 74.3  (62.5, 84.6) | 0.48 |
| Comorbidities |  |  |  |  |
| Hypertension | 47 (81) | 67 (92) | 114 (87) | 0.11 |
| Diabetes | 7 (12.1) | 14 (19.2) | 21 (16) | 0.34 |
| Coronary heart disease | 10 (17.2) | 19 (26) | 29 (22.1) | 0.29 |
| Smoking^1^ | 9 (15.5) | 7 (9.9) | 16 (12.4) | 0.42 |
| Etiologies of kidney disease |  |  |  | 0.29 |
| Glomerulosclerosis | 9 (15.5) | 21 (28.8) | 30 (22.9) |  |
| ADPKD | 14 (24.1) | 12 (16.4) | 26 (19.8) |  |
| Glomerulonephritis | 11 (19) | 12 (16.4) | 23 (17.6) |  |
| Diabetes | 5 (8.6) | 10 (13.7) | 15 (11.5) |  |
| Previous graft failure | 4 (6.9) | 4 (5.5) | 8 (6.1) |  |
| Reflux/pyelonephritis | 1 (1.7) | 4 (5.5) | 5 (3.8) |  |
| Congenital | 2 (3.4) | 1 (1.4) | 3 (2.3) |  |
| Interstitial nephritis | 3 (5.2) | 0 | 3 (2.3) |  |
| Other^2^ | 9 (15.5) | 9 (12.3) | 18 (13.7) |  |
| Induction immunosuppression |  |  |  |  |
| Basiliximab | 44 (75.9) | 11 (15.1) | 55 (42) | **<0.001** |
| Thymoglobulin | 13 (22.4) | 73 (100) | 86 (65.6) | **<0.001** |
| Maintenance immunosuppression |  |  |  |  |
| Ciclosporine | 18 (31) | 10 (13.7) | 28 (21.4) | **0.01** |
| Tacrolimus | 30 (51.7) | 56 (76.7) | 86 (65.6) | **0.003** |
| MMF | 37 (63.8) | 31 (42.5) | 68 (51.9) | **0.02** |
| mTOR | 2 (3.4) | 1 (1.4) | 3 (2.3) | 0.58 |
| Cold ischemia time (min)  median (IQR) | 328  (97.8, 612) | 555  (95, 783) | 415  (96,726) | 0.11 |
| Previous renal graft | 7 (12.1) | 11 (15.1) | 18 (13.7) | 0.8 |
| Number of previous grafts |  |  |  | 0.84 |
| 1 | 51 (87.9) | 62 (84.9) | 113 (86.3) |  |
| 2 | 5 (8.6) | 9 (12.3) | 14 (10.7) |  |
| >2 | 2 (3.4) | 2 (2.7) | 4 (3.1) |  |
| Dialysis prior to transplant | 33 (56.9) | 44 (60.3) | 77 (58.8) | 0.72 |
| Dialysis type prior to transplant^3^ |  |  |  | 0.16 |
| HD | 24 (72.7) | 38 (86.4) | 62 (80.5) |  |
| PD | 9 (27.3) | 6 (13.6) | 15 (19.5) |  |
| Donor type |  |  |  | **0.03** |
| DBD | 25 (43.1) | 31 (42.5) | 56 (42.7) |  |
| Living | 28 (48.3) | 23 (31.5) | 51 (38.9) |  |
| DCD | 5 (8.6) | 19 (26) | 24 (18.3) |  |
| Donor |  |  |  |  |
| Sex, Female | 36 (62.1) | 39 (53.4) | 75 (57.3) | 0.38 |
| Age (Years)  Median (IQR) | 57.5  (48, 63) | 54  (45, 62) | 55  (47, 63) | 0.19 |
| Kidney dysfunction post-transplant |  |  |  | 0.07 |
| DGF | 10 (17.2) | 23 (31.5) | 33 (25.2) |  |
| PNF | 1 (1.7) | 0 | 1 (0.8) |  |

ADPKD autosomal dominant polycystic kidney disease, BMI body mass index, DBD donor after brain death, DCD donor after cardiac death, DGF delayed graft function, HD hemodialysis, MMF mycophenolate mofetil, mTOR mammalian target of rapamycin, PD peritoneal dialysis, PNF primary non function.

^1^ There were 5 missing values

^2^ Other etiologies included nephrocalcinosis, thrombotic microangiopathy, acute kidney injury post sepsis, eclampsia, cortical necrosis or unknown

^3^ There were 67 missing values

**Supplement Table 2.** Weekly valganciclovir prophylaxis dosing according to renal function (reported in week-entries).

1. Weeks 0-4

|  | **D+R-**  n=176 (%) | **R+/TG**  n=214 (%) | **Total**  n=390 (%) | ***p* value** |
| --- | --- | --- | --- | --- |
| **VGC dose assessment** | | | | 0.05 |
| Appropriately dosed | 84 (47.7) | 81 (37.9) | 165 (42.3) |  |
| Inappropriately dosed | 92 (52.3) | 133 (62.1) | 225 (57.7) |  |
| Daily VGC dose in mg  median (IQR) | 450  (450, 900) | 450  (450, 900) | 450  (450, 900) | 0.75 |
| Creatinine (umol/l)  median (IQR) | 127  (105, 154) | 134  (106, 166) | 130  (105, 163) | 0.15 |
| eGFR (ml/min/1.73m^2^)  median (IQR) | 51  (39, 62) | 46  (34, 60) | 48  (36, 60) | 0.02 |

1. Weeks 5-24

|  | **D+R-**  n=356 (%) | **R+/TG**  n=286 (%) | **Total**  n=642 (%) | ***p* value** |
| --- | --- | --- | --- | --- |
| **VGC dose assessment** | | | | 0.3 |
| Appropriately dosed | 157 (44.1) | 138 (48.3) | 295 (46) |  |
| Inappropriately dosed | 199 (55.9) | 148 (51.7) | 347 (54) |  |
| Daily VGC dose in mg  median (IQR) | 450  (450, 900) | 450  (450, 450) | 450  (450, 450) | 0.02 |
| Creatinine (umol/l)  median (IQR) | 137  (112, 164) | 129  (106, 157) | 132  (108, 158) | 0.05 |
| eGFR (ml/min/1.73m^2^)  median (IQR) | 50  (39, 60) | 48  (38, 60) | 49  (38, 60) | 0.25 |

eGFR estimated Glomerular Filtration Rate, D Donor, R Recipient, TG Thymoglobulin, VGC Valganciclovir.

**Supplement Table 3.** Clinically significant (cs) CMV inrection and type of organ involvement among all first breakthrough csCMV-infections

|  | **D+R-**  n=8 (%) | **R+/TG**  n=11 (%) | **Total**  n=19 (%) | ***p* value** |
| --- | --- | --- | --- | --- |
| Primary infection | 8 (100) | 0 | 8 (42.1) | <0.001 |
| Reactivation | 0 | 11 (100) | 11 (57.9) |  |
| **Symptomatology** | | | |  |
| Asymptomatic | 5 (62.5) | 11 (100) | 16 (84.2) | 0.06 |
| Probable disease^1^ | 1 (12.5) | 0 | 1 (5.3) |  |
| Undefined | 2 (25) | 0 | 2 (10.5) |  |

D Donor, R Recipient, TG Thymoglobulin.

^1^Probable liver CMV disease

**Supplement Table 4.** Sensitivity analysis with additional random slope for FUP weeks, but same predictors of cytopenias on multivariable analysis.

| Lymphopenia | OR | 95% CI | *p* value |
| --- | --- | --- | --- |
| VGC overdosing | 6.65 | 1.55 – 28.56 | 0.011 |
| MMF* | 8.48 | 0.12 – 579.5 | 0.32 |
| FUP | 2.35 | 0.72 – 7.67 | 0.16 |
|  |  |  |  |
| Leucopenia |  |  |  |
| VGC overdosing | 2.92 | 0.55 – 15.65 | 0.21 |
| MMF* | 3.06 | 0.22 – 42.61 | 0.40 |
| FUP | 1.19 | 0.87 – 1,61 | 0.28 |
|  |  |  |  |
| Neutropenia |  |  |  |
| VGC overdosing | 1.65 | 0.30 – 8.89 | 0.57 |
| MMF* | 0.48 | 0.04 – 5.67 | 0.56 |
| FUP | 1.23 | 1.11 – 1.37 | <0.001 |
|  |  |  |  |
| Thrombocytopenia |  |  |  |
| VGC overdosing | 0.59 | 0.11 – 3.14 | 0.53 |
| MMF* | 1.03 | 0.01 – 151.1 | 0.99 |
| FUP | 0.94 | 0.78 – 1.13 | 0.50 |

FUP follow up weeks, MMF mycophenolate mofetil, OR odds ratio, VGC valganciclovir, 95% CI 95% confidence interval.

^*^Overall, 19/105 MMF treatment durations had a missing stopdate, and the duration was imputed via median.
